# Identification of a novel SDHB c.563 T > C mutation responsible for Paraganglioma syndrome and genetic analysis of the SDHB gene in China: a case report

**DOI:** 10.1186/s12881-020-01049-3

**Published:** 2020-05-27

**Authors:** Heye Chen, Wei Yao, Qing He, Xuefang Yu, Bo Bian

**Affiliations:** 1 Department of Endocrinology and Metabolism, Tianjin Medical University General Hospital, Tianjin, 300070 China; 2 Department of Cardiology, Tianjin Medical University General Hospital, Tianjin, 300070 China

**Keywords:** Pheochromocytoma, Paraganglioma, Succinate dehydrogenase, Mutation, Metastasis, Case report

## Abstract

**Background:**

Pheochromocytoma/paraganglioma (PPGL) is a rare neuroendocrine tumor. Succinate dehydrogenase (SDH) deficiency has been confirmed to be associated with PPGL in various studies. SDHB mutations play an important role in PPGL. However, genetic screening of PPGL patients has not been widely carried out in clinics in China, and only a few related studies have been reported.

**Case presentation:**

We report a case of a 23-year-old woman with paraganglioma (PGL) caused by a novel missense SDHB mutation, c.563 T > C (p.Leu188Pro), who presented with paroxysmal hypertension. Computed tomography (CT) and magnetic resonance imaging (MRI) revealed a PGL in the right retroperitoneum and no metastasis. The patient was treated with surgical excision and did not have postsurgerical paroxysmal hypertension. In addition, we searched the literature related to variations in SDHB genes in Chinese patients with PPGL using multiple online databases, including PubMed, China Hospital Knowledge Database and Wanfang Data. Ultimately, 14 studies (published between 2006 and 2019) comprising 34 cases of SDHB-related PGL or pheochromocytoma (PCC) were found. In total, 35 patients were enrolled in this study, and 25 mutations were identified. The common genetic alterations of SDHB in China were c.136C > T (11.4%), c.18C > A (11.4%) and c.725G > A (8.5%). Some carriers of SDHB mutations (28.1%) developed metastatic PPGL, and a high frequency of head and neck PGLs (HNPGLs) (59.4%) was reported.

**Conclusions:**

We describe a classic case with a novel SDHB c.563 T > C mutation. Based on our literature review, common SDHB gene mutations in Chinese PPGL patients are c.136C > T, c.18C > A and c.725G > A.

## Background

Paraganglioma (PGL) and pheochromocytoma (PCC) are also referred to as pheochromocytoma/paraganglioma (PPGL), which are rare neuroendocrine tumors. These tumors may secrete catecholamines, which may cause paroxysmal hypertension, palpitations, headache and diaphoresis and may eventually lead to serious cardiovascular complications.

Recent studies have shown that approximately 1/3 of PPGL patients have a genetic background [1]. It has also been reported that approximately 40% of all PGLs and 3% of all PCCs are associated with succinate dehydrogenase (SDH) deficiency [2]. SDH, which is a respiratory enzyme, plays a key role that links the Krebs cycle and the electron transport chain, and SDH is regulated by SDHA, SDHB, SDHC and SDHD [3]. SDHB, which contains two highly conserved L(I)YR motifs, is the Fe-S subunit of complex II [4]. The two L(I)YR motifs are necessary for Fe-S clusters via recruitment of the Fe-S transfer machinery [4].

In many cases, SDHB-related disease is characterized by a single tumor [5], and carriers of gene variants commonly develop extra-adrenal PGLs, PCCs and metastatic disease than do carriers of mutations in the other SDH subunits [6–8]. In addition, SDHB-related PPGLs are reported to be associated with malignancy rates as high as 7.7–97% [6–12].

At present, genetic screening of PPGL patients has not been widely carried out in Chinese clinics, and only a few related studies have been conducted. The aims of the study are to report a novel SDHB c.563 T > C mutation and to investigate SDHB variations in Chinese PPGL patients. Therefore, we collected all literature related to SDHB variations in PPGL in Chinese people.

## Case presentation

A 23-year-old female presented with complaints of paroxysmal hypertension (the highest BP was 230/180 mmHg) with palpitations, headache, diaphoresis and vomiting for 11 months. All of her sudden hypertension attacks were treated with antihypertensive drugs. Three days prior, the patient presented to the emergency department again with paroxysmal hypertension (BP 173/139 mmHg) and the above symptoms, but obvious abnormalities were not found on physical examination. One year prior, she had undergone laparoscopic cholecystectomy for gallstones. In addition, she had no history of other systemic diseases.

After an extensive workup, the patient was found to have elevations of plasma methoxynorepinephrine and urine vanillylmandelic acid, but her plasma metanephrine level was normal (Table 1).

**Table 1:** Biochemical characteristics

	Data	Reference range
metanephrine (nmol/l)	< 0.08	≤0.5
methoxynorepinephrine (nmol/l)	> 20.56	≤0.90
urine vanillylmandelic acid (μmol/24 h)	119.9265	< 68.60

**Fig. 1:**
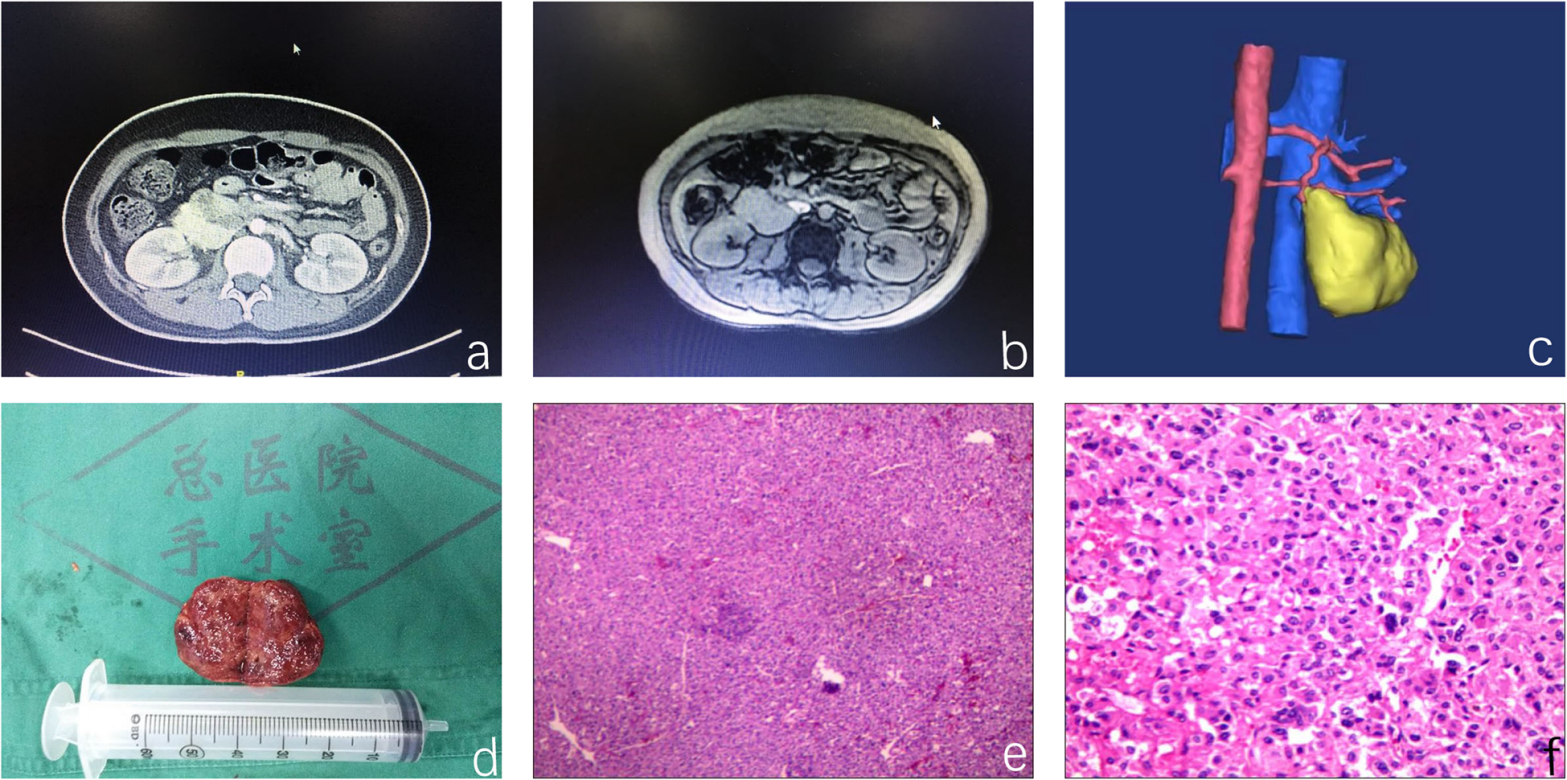
**a**-**c** CT and MRI showed a 4.6 × 3.1 cm retroperitoneal mass on the right retroperitoneum, and the boundary between the mass and the IVC was not clear; **d**-**f** Pathology and immunohistochemical staining: Syn and CgA were positive, Melan A, HMB45 and α-inhibin were negative, S-100 cells were positive, and the CD31 vascular endothelium marker was positive

Subsequent CT and MRI showed a 4.6 × 3.1 cm retroperitoneal mass on the right retroperitoneum, and the boundary between the mass and the inferior vena cava (IVC) was not clear (Fig. 1). Enhanced CT scanning of the thorax, abdomen and pelvic cavities showed no metastasis. Before admission, the patient had undergone cervical CT because of the symptoms mentioned above, and the results were normal. However, considering the clinical history and inapparent bilateral adrenal glands, we favored the clinical diagnosis of retroperitoneal PGL.

The patient was given doxazosin and metoprolol for 2 weeks as preoperative preparation. Then, the patient was medically managed with surgical excision. Immunohistochemical staining: Syn and CgA were positive, Melan A, HMB45 and α-inhibin were negative, S-100 cells were positive, and the CD31 vascular endothelium marker was positive. Conclusion: right retroperitoneal PGL (Fig. 1). However, the local capsule of the tumor was incomplete.

**Fig. 2:**
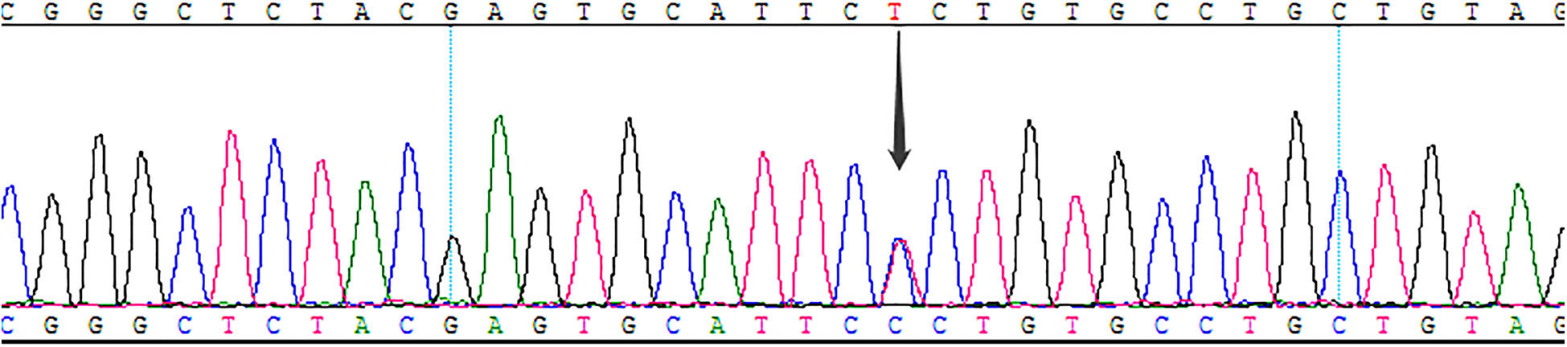
The novel variant of the SDHB gene: Genomic DNA analysis of peripheral blood leukocytes, showing a germline missense mutation in exon 6 of the SDHB gene [c.563 T > C] (arrow)

To further determine the cause of the disease, we performed genetic testing with consent from the patient. Genetic testing demonstrated that the patient carried a missense mutation in exon 6 of the SDHB gene [c.563 T > C] (Fig. 2). The identified mutation was classified as likely pathogenic (class 1). This variation is novel, and there are no relevant research reports at present. Since the patient is an orphan, we could not obtain her pedigree for the SDHB-linked family.

Clinical follow-up examinations were carried out three times through telephone interviews or outpatient visits. One year after surgery, the patient did not exhibit paroxysmal hypertension (BP 90–110/60–70 mmHg) or the symptoms described above. Meanwhile, an abdominal CT scan did not indicate any masses. However, it will be necessary to perform long-term follow-up and screening of this patient over her lifetime.

## Discussion and conclusion

With widespread PPGL genetic testing, the clinical manifestations of many PPGL-related genes have become well understood. Our study reports a novel SDHB c.563 T > C mutation. To date, Human Gene Mutation Database (HGDM, http://www.hgmd.cf.ac.uk/) includes 254 SDHB gene mutations, but the c.563 T > C variant has not been reported. This specific case adds to our knowledge of PCCs and PGLs and may help with genetic counseling of patients.

However, genetic screening of PPGL patients has not been widely carried out in Chinese clinics, and few related studies have been conducted. Therefore, to analyze and evaluate the variations of SDHB genes in Chinese patients with PPGL, we carried out a systematic literature review using multiple online databases, including China Hospital Knowledge Database (CNKI) (http://www.chkd.cnki.net), Wanfang Data (http://www.wanfangdata.com.cn/),and PubMed (https://www.ncbi.nlm.nih.gov/pubmed), by using the key words “SDHB,” and “China”. The references listed in the relevant studies were carefully screened to identify additional studies. In total, 15 studies (published between 2006 and 2019) were identified (Table 2), comprising 35 cases (including the current case) of SDHB-related PGL or PCC .

**Table 2:** Characterization of Fifteen Related Studies on SDHB Mutations in PPGL Patients

Reference	Year	Age	Sex	Exon	cDNA	Protein	Type	PGL/PCC	Location	Malignant disease
[13]	2006	32	F	7	c.689G > A	p.R230H	Missense	PGL	Para-aortic abdominal	No
		17	M	7	c.757delT	p.C253Vfs257X	Frameshift	PGL	Middle mediastinum	Yes
[14]	2007	22	F	7	c.640C > T	p.Q214X	Nonsense	PCC^a^	Left adrenal gland	No
[15]	2009	15	F	2	c.136C > T	p.R46X	Nonsense	PGL^a^	Postcaval abdominal	No
		39	F	3	c.268C > T	p.R90X	Nonsense	PGL	Para-aortic abdominal	Yes
		22	F	7	c.725G > A	p.R242H	Missense	PCC	Right adrenal gland	No
[16]	2010	53	F	3	c.269G > A	p.R90E	Missense	PGL	HN/RCBT	No
		36	F	6	c.597C > G	p.Y199X	Nonsense	PGL	HN/RCBT	No
		43	F	7	c.709C > T	p.P237S	Missense	PGL	HN/RCBT	No
		31	F	2	c.200 + 1G > C	p.?	Splice site	PGL	HN/RGJT	No
		29	F	1	c.20-22delinsC	p.L7PrfsX55	Frameshift	PGL	HN/RGJT	Yes
		31	F	7	c.725G > A	p.R242H	Missense	PGL	HN/RGJT	No
		37	F	7	c.725G > A	p.R242H	Missense	PGL	HN/RGTT	No
		33	M	2	c.79C > A	p.R27X	Nonsense	PGL	HN/LCBT	No
		38	F	6	c.597C > G	p.Y199X	Nonsense	PGL	HN/RCBT	No
		30	F	2	c.137G > A	p.R46Q	Missense	PGL	HN/LCBT	No
[17]	2010	NA	NA	6	c.591del C	p.S198Afs219X	Frameshift	NA	NA	NA
		NA	NA	7	c.688C > T	p.R230C	Missense	NA	NA	NA
[18]	2011	NA	NA	2	c.136C > T	p.R46X	Nonsense	NA	NA	NA
[19]	2013	58	M	6	c.595C > A	p.S195R	Missense	PGL	HN	No
		30	F	1	c.18C > A	p.A6A	Synonymous	PGL	HN	Yes
		17	M	1	c.18C > A	p.A6A	Synonymous	PGL	HN	Yes
		47	F	6	c.595C > A	p.S195R	Missense	PGL	HN	No
		29	F	1	c.18C > A	p.A6A	Synonymous	PGL	HN	Yes
		37	M	1	c.18C > A	p.A6A	Synonymous	PGL	HN	No
[20]	2014	30	M	4	c.380 T > G	p.I127S	Missense	PGL^b^	Abdominal→HN	No
[21]	2015	30	NA	2	c.112delC	p.R38Vfs77X	Frameshift	PGL	Bladder	Yes
[22]	2015	54	M	7	c.647A > G	p.Y216C	Missense	PGL	HN	No
		38	M	–	Del exon 1,2,3,7,8	–	Large deletion	PGL	HN	Yes
[23]	2018	14	M	4	c.343C > T	p.R115X	Nonsense	PGL	Postcaval abdominal	No
		32	M	5	c.541-542A > G	IVS5-2A > G	Splice site	PGL	Para-aortic abdominal	No
[24]	2018	46	M	2	c.136C > T	p.R46X	Nonsense	PCC	Right adrenal gland	Yes
[25]	2018	12	F	2	c.136C > T	p.R46X	Nonsense	PGL	Upper left mediastinum	No
[26]	2019	16	F	4	c.423 + 1G > T	p.?	Splice site	PGL	Retroperitoneal	No
Current case	2019	23	F	6	c.563 T > C	p.L188P	Missense	PGL	Retroperitoneal	No

*CBT* Carotid body tumor, *GJT* Glomus jugulare tumor, *GGT* Glomus tympanicum tumor, *NA* Not applicable.

^a^ palindromic tumors

^b^ multiple tumors

The patients included 35.4% (11/31) males and 64.5% (20/31) females, and the mean age at first evaluation was 31.9 ± 11.9 years (range: 12–58 years). Of the 35 patients diagnosed with PPGL, 54.5% (18/33) of primary tumors were in the head and neck, 9.1% (3/33) were in the adrenal gland, and 33.4% (11/33) were in an extra-adrenal gland. In addition, 9/32 (28.1%) carriers of SDHB mutations developed metastatic PPGL, including 5 cases of head and neck paragangliomas (HNPGLs), 1 case of PCC and 3 cases of extra-adrenal sympathetic paraganglioma (sPGL). Although previous studies have shown much higher rates for the development of sPGLs (approximately 60%) [7, 12, 27], the frequency of HNPGLs among SDHB mutation carriers was high in our study, at approximately 59.4%. Recently, French [28] and Dutch [11] groups published mutation studies of SDHB with proportions similar to those reported in our study, and the prevalence rates of PCC and sPGLs in their studies were 1.6 and 6.5% or 2.1 and 13.4%, respectively. There was a high proportion of index patients in previous studies, which could lead to ascertainment bias and underestimation of the proportion of HNPGLs. In addition, our review includes three HNPGL studies, which may increase the proportion of HNPGLs among SDHB mutation carriers.

In our study, 9/32 (28.1%) SDHB mutation carriers developed metastatic PGL/PCC, which included 5 cases of HNPGLs, 1 case of PCC and 3 cases of sPGLs. The rate of metastatic disease was lower than that reported in previous studies [6, 8, 9, 12]. Some have proposed that selection bias in referral-based studies is a major reason for a very high rate of malignant PGL in SDHB mutation carriers. In addition, we suggest that recurrent and malignant tumors might occur years after primary PPGL surgery; thus, the prevalence of recurrence and malignancy may be underestimated. In other words, the discrepancy in malignancy rates may be linked to the different follow-up times.

In addition, for HNPGL patients, the rate of metastatic diseases was 15.6% (5/32), which was higher than the rates observed for sPGL and PCC patients. Therefore, patients with HNPGL have a high malignancy risk. Moreover, a recent study reported that patients with SDH mutation have a higher risk of later development of metachronous tumors and recurrence than do patients without mutation in this gene [29]. In summary, radiological screening is very important among carriers of SDHB mutations, and follow-up of those patients, especially the head and neck region, should be undertaken.

Of the 35 SDHB gene variants, we found 25 different mutations, and SDHB pathogenic mutations included missense mutations (*n* = 10), nonsense mutations (*n* = 6), frameshift mutations (*n* = 4), splice site mutations (*n* = 3), synonymous mutations (n = 1), and deletions of one or more exons (n = 1). Common genetic alterations of SDHB in Chinese patients included c.136C > T (11.4%), c.18C > A (11.4%) and c.725G > A (8.5%). The c.136C > T (p.R46X) mutation and the c.725G > A (p.R242H) mutation occur in the first highly conserved (I44Y45R46) motif of SDHB and the second (L240Y241R240) motif, which are essential for incorporation of the Fe-S cluster into SDHB [4, 30]. Fe-S clusters are vitally important to electron transport and function, and this mutation completely abrogates SDH activity. However, the c.18C > A (p.A6A) mutation is a synonymous mutation, and 3/4 of carriers of this variation have metastatic disease. Thus, we suggest that c.18C > A may be one of the phenotypic causes of HNPGLs.

Interestingly, in our results, three frameshift mutations (c.757delT, c.20-22delinsC and c.112delC) were associated with metastatic disease. The term frameshift mutation refers to a change of the reading frame, resulting in the original gene encoding one peptide chain and the variant gene encoding a completely different peptide chain sequence. This change may render PPGL caused by frameshift mutations prone to metastasis, which highlights the necessity of follow-up for those patients.

Finally, our results have some limitations. On the one hand, few related studies have been performed in China, and some studies that lacked complete data were excluded. This inevitably led to limited case collection, which could lead to unreliable results. On the other hand, we did not perform genomic analysis of family members, which limits our ability to assess the association of PPGL morbidity with SDHB mutations. Moreover, without functional studies, we cannot determine the true pathogenicity of SDHB mutations.

In conclusion, we report a novel SDHB c.563 T > C mutation and investigate SDHB mutations among PPGL patients in China in this literature review. Thus, it is necessary to develop genetic screening for PPGL patients to guide diagnosis, treatment and follow-up. Large studies of SDHB mutations are needed to analyze the characteristics of these patients in China.

## Data Availability

The sequence datasets generated during the current study are not publicly available because it is possible that individual privacy could be compromised.

## Abbreviations

PPGL: Pheochromocytoma/paraganglioma
PGL: Paraganglioma
PCC: Pheochromocytoma
SDH: Succinate dehydrogenase
IVC: Inferior vena cava
CT: Computed tomography
MRI: Magnetic resonance imaging
HGDM: Human Gene Mutation Database
CNKI: China Hospital Knowledge Database
HNPGLs: Head and neck paragangliomas
sPGLs: Extra-adrenal sympathetic paragangliomas
